# Energy budget diagnosis of changing climate feedback

**DOI:** 10.1126/sciadv.adf9302

**Published:** 2023-04-21

**Authors:** B. B. Cael, Jonah Bloch-Johnson, Paulo Ceppi, Hege-Beate Fredriksen, Philip Goodwin, Jonathan M. Gregory, Christopher J. Smith, Richard G. Williams

**Affiliations:** ^1^ National Oceanography Centre, Southampton, UK.; ^2^ National Centre for Atmospheric Science, Reading, UK.; ^3^ Imperial College London, London, UK.; ^4^ UiT The Arctic University of Norway, Tromsø, Norway.; ^5^ University of Southampton, Southampton, UK.; ^6^ Met Office Hadley Centre, Exeter, UK.; ^7^ University of Leeds, Leeds, UK.; ^8^ International Institute for Applied Systems Analysis, Laxenburg, Austria.; ^9^ University of Liverpool, Liverpool, UK.

## Abstract

The climate feedback determines how Earth’s climate responds to anthropogenic forcing. It is thought to have been more negative in recent decades due to a sea surface temperature “pattern effect,” whereby warming is concentrated in the western tropical Pacific, where nonlocal radiative feedbacks are very negative. This phenomenon has however primarily been studied within climate models. We diagnose a pattern effect from historical records as an evolution of the climate feedback over the past five decades. Our analysis assumes a constant rate of change of the climate feedback, which is justified post hoc. We find a decrease in climate feedback by 0.8 ± 0.5 W m^−2^ K^−1^ over the past 50 years, corresponding to a reduction in climate sensitivity. Earth system models’ climate feedbacks instead increase over this period. Understanding and simulating this historical trend and its future evolution are critical for reliable climate projections.

## Teaser

Energy budget records indicate that Earth's climate feedback has become substantially more negative over the past 50 years.

## INTRODUCTION

Earth’s climate feedback—the amount of extra energy radiated to space per degree of global warming [λ, (W m^−2^ K^−1^), a negative number]—is a central object of study in climate science, being one of the essential parameters determining Earth’s response to anthropogenic emissions of greenhouse gases and other forcing agents (*1*). If λ is more negative, Earth’s global mean surface temperature *T* (K) is less sensitive to the anthropogenic radiative forcing *F* (W m^−2^), i.e., −λ is inversely proportional to effective climate sensitivity (defined as the projected equilibrium warming following a doubling of the preindustrial atmospheric CO_2_ concentration) (*2*). Although myriad physical processes contribute to λ, a crucial factor is the spatial pattern of warming. In particular, warming in the western tropical Pacific produces a much larger global radiative response and, hence, a more negative climate feedback than warming elsewhere. This phenomenon has been termed the “pattern effect” (*3*–*7*). Warming in this region, where air moves upward in the lower atmosphere, results in increased stability of the lower tropical atmosphere in remote subsidence regions. This far-field adjustment in turn increases atmospheric stability and low cloud cover and hence upward shortwave radiation. In recent decades, global warming has been concentrated in this Western Pacific region of very negative radiative feedbacks (*4*, *5*, *8*–*13*), leading to a more negative value of λ (hence, lower climate sensitivity). More generally, it is the east-west tropical Pacific surface temperature gradient that is of particular importance (*14*) and emphasizes that the recent cooling in the eastern equatorial Pacific is important in setting λ.

When simulating the historical climate over recent decades, Earth system models (ESMs) tend to produce spatial warming patterns that lack this concentration of warming in regions of very negative radiative feedbacks. The ESM projections lead to less negative λ (higher climate sensitivity) than when the observed spatial pattern of sea surface temperature is imposed on the same atmospheric models (*11*). Furthermore, for standard ESM simulations with fixed atmospheric CO_2_ concentrations quadrupling from preindustrial levels—the primary model experiment for diagnosing the global climate feedback λ—the spatial pattern of warming is again quite different, leading to a less negative λ than indicated by observations (*15*). The pattern effect is thus sometimes quantified by the difference between the λ values associated with a CO_2_-quadrupling experiment and an experiment with prescribed sea surface temperatures (*9*). This equivalence is based on the argument that the surface warming should eventually adjust to the modeled long-term warming pattern. The pattern effect quantified as such has been almost exclusively studied within ESMs, most notably using Green’s functions (*5*) or comparing different model experiments (*11*).

It would be advantageous to quantify a pattern effect from historical records to assess the probability, magnitude, and implications of this effect for Earth’s recent climate and to provide a benchmark with which to assess ESM performance. This observationally based viewpoint is especially important because it is increasingly common to weight models in multimodel projections by their relative performance in capturing historical trends (*16*–*19*). If these weighting schemes do not account for the influence of the pattern effect, the resulting model-averaged response may produce biased projections because models may capture historical trends for the wrong reasons.

Here, we propose an alternative metric for the pattern effect—the trend in the climate feedback λ over recent decades—that can be diagnosed from historical records without reference to hypothetical scenarios. This approach is based on an integral equation and therefore estimates an effective climate feedback (*20*). We show that the trend we diagnose is significantly different from zero over the past five decades of global energy budget records and large in amplitude with substantial implications for global warming. We also show that ESMs fail to capture this trend, irrespective of their climate sensitivity. We use the past five decades because this is the time period over which reliable records exist (Methods) (*21*). The bulk of global warming has occurred since 1970, with four of the first 6 years of the 1970s being within 0.2°C of the 1850–1900 average (*22*). The bulk of the increase in ocean heat content H (W y m^−2^) and radiative forcing (*21*, *23*) has also occurred since 1970. (Note that throughout this paper, script letters indicate a time integral; here, ocean heat content change is the time integral of the rate of ocean heat uptake.) We stress from the outset that we only investigate this period of 1970–2019; we do not make any assumptions or speculations about the future trends in λ.

## RESULTS AND DISCUSSION

The method we present is described in detail in Methods—the reader interested in the full derivation should read the “Theory” section before proceeding further. Briefly, we work from a simple Earth energy balance equation, which states that the rate of global warming is proportional to the net rate of energy storage in the upper ocean

$$\eta \dot{T}(t)=F(t)+\lambda (t)T(t)-H(t)$$ (1)

where *T* (K) is the global mean surface temperature anomaly, η (W y m^−2^ K^−1^) is the heat capacity of the upper ocean layer, *F* (W m^−2^) is the radiative forcing imposed upon Earth’s surface, λ (W m^−2 ^K^−1^) is the climate feedback, and *H* (W m^−2^) is the rate of ocean heat uptake (the flux of energy into the deeper ocean from the upper layer). From this equation, one can derive the energy budget F(τ) − H(τ) = R(τ), where F(τ) is the cumulative energy fluxed to the top of the atmosphere via radiative forcing by time τ; H(τ) is the ocean heat content anomaly at time τ (including the upper layer), which approximates the Earth’s energy imbalance; and R(τ) is the cumulative energy fluxed back to space by time τ; this is just a restatement of conservation of energy. We then make the ansatz that λ changes linearly with time from 1970, i.e., λ(*t*) = λ_1970_(1 + μ*t*), where *t* is time in years since 1970 and μ (y ^−1^) is the annual rate of change in λ relative to the initial value λ_1970_ {i.e., $\mu =\frac{1}{\text{Δ}t}\left[\frac{\lambda (1970+\text{Δ}t)}{\lambda_{1970}}-1\right]$ for some number of years Δ*t*}. This ansatz is justified post hoc by the absence of systematic behavior in the residuals (Methods). The choice of a linearly changing λ is motivated by its simplicity as a means to capture the expected change in λ from 1970 to 2019 as warming concentrated in the western tropical Pacific. The term λ_1970_ × μ (W m^−2^ K^−1^ y^−1^) captures the average annual rate of change in λ over this period. An approximately linear evolution in λ over this period could arise for multiple reasons, but arguably the most plausible is the convolution of a changing warming pattern with spatial variation in regional climate feedbacks (*24*). If there is sufficient spatial variation in the feedback per warming in different locations (*5*), and the spatial pattern of warming changes over time (*25*), then the area-integrated global effective feedback parameter λ will likewise evolve over time, with its value depending on how much warming occurs in places with more or less negative feedbacks. If this warming pattern evolves gradually (*25*), then the change in the effective feedback parameter λ on a 50-year time scale will be approximately linear in time.

**Fig. 1.**
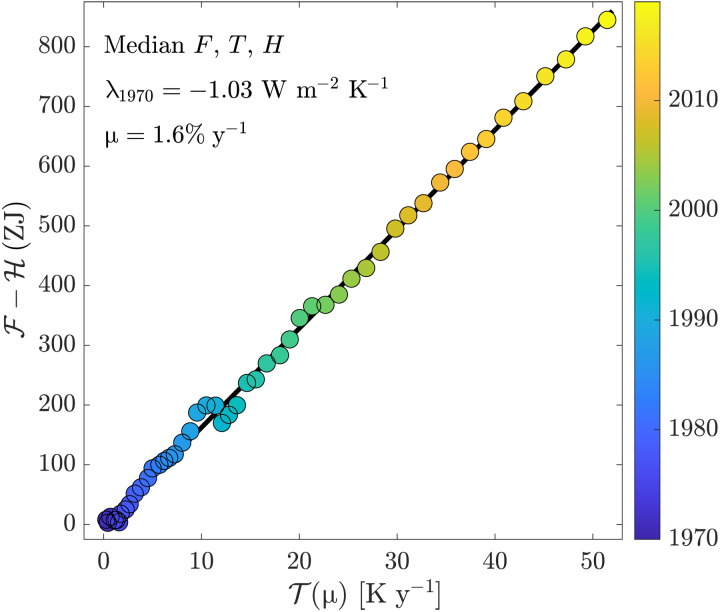
Illustration of diagnosis of model parameters from time series of integral quantities using Eq. 2 and the medians of global area-weighted mean radiative forcing, surface temperature, and ocean heat content (see Methods for details). *x* axis is the time-weighted temperature anomaly integral. *y* axis is the cumulative anomaly in energy radiated to space (F − H). Color indicates the year.

Substituting this ansatz into the energy budget yields

$$F(\tau )-H(\tau )=-\lambda_{1970}T(\mu ,\tau )$$ (2)

where T(μ, τ) is a weighted integral of the temperature anomaly (Methods). The parameter combinations (λ_1970_, μ) that minimize the residuals of this equation are selected. Ensembles for F, T, and H are used to quantify uncertainty; the HadCRUT5 (*22*) global annual mean surface temperature *T* product (with different dataset realizations that sample the measurement uncertainty), the *F* ensemble from the recent Working Group I contribution to the Intergovernmental Panel on Climate Change’s Sixth Assessment Report (*23*), and an H ensemble generated from three observational ocean heat content products are used (*26*–*28*). Figure 1 shows a regression that illustrates this process for the median radiative forcing and temperature anomaly, as well as the associated parameter values. Heuristically, we choose the μ that makes the relationship between T(μ) and F − H most linear. The slope of this linear relationship then corresponds to λ_1970_. (In comparison for a choice of μ = 0, there is significant curvature in this relationship; see fig. S1.)

Within this framework, we begin by testing the null hypothesis of a constant climate feedback from 1970 to 2019, i.e., μ = 0. We reject this hypothesis for three reasons. When we fit our statistical model with μ = 0 to the historical records, 92% of ensemble members yield curvature of the same sign in the residuals, indicating systematic behavior not captured by a constant climate feedback (fig. S2 and Methods). When we compare the μ = 0 model with a model with a nonzero μ, 91% of ensemble members yield higher Akaike information criterion (AIC) values for the μ = 0 model (fig. S2 and Methods), indicating that a time-varying climate feedback describes these data better even after penalizing for the additional free parameter. Last, when μ is allowed to be nonzero, we find a decreasing climate feedback trend for 92% of ensemble members (fig. S2).

**Fig. 2.**
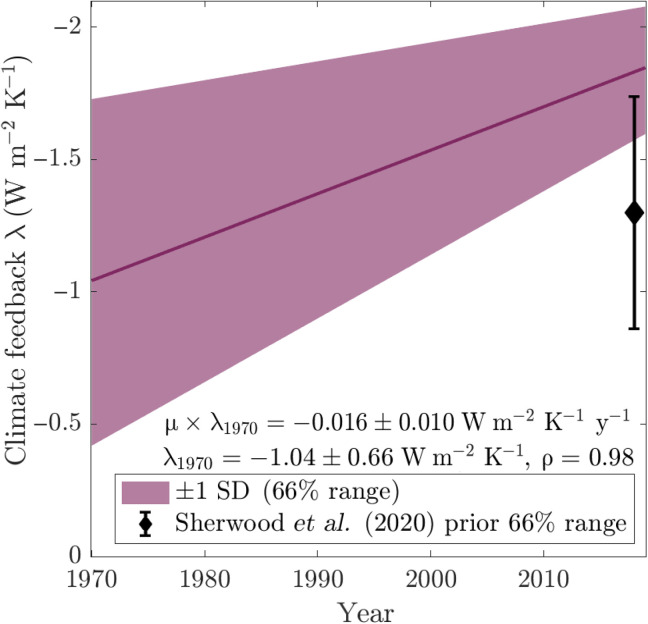
Median and 66% range (∼±1 SD) of the climate feedback λ as estimated from Eq. 2. The black error bar represents the estimated feedback corresponding to a doubling of CO_2_ (*9*).

Our analysis thus suggests that λ became more negative with time (decreasing climate sensitivity) over the period 1970–2019 (i.e., μ > 0). Figure 2 shows our main result; we find that λ has decreased by 0.8 ± 0.5 W m^−2^ K^−1^ (± indicates half of 66% range or ∼1 SD throughout) from −1.0 ± 0.7 W m^−2^ K^−1^ in 1970 to −1.8 ± 0.2 W m^−2^ K^−1^ in 2019. This corresponds to an annual decrease of μ × λ_1970_ = 0.016 ± 0.010 W m^−2^ K^−1^ y^−1^. The reduced uncertainty in the 2019 values is because uncertainties in μ and λ_1970_ are strongly correlated (Spearman rank correlation of 0.98) because observational uncertainties, and hence uncertainties in the climate feedback, reduce with time. This is a large change from a λ estimate that moves from the low end of a priori expectations [−1.3 ± 0.44W m^−2^ K^−1^ (*9*); Fig. 2] in 1970 to the high end in 2019. Our estimate of the change over this period of 0.8 ± 0.5 W m^−2^ K^−1^ is consistent with the ESM-based quantifications of the pattern effect of 0.5 ± 0.5 W m^−2^ K^−1^ (*9*) or 0.6 W m^−2^ K^−1^ with a range of 0.3 to 1.0 W m^−2^ K^−1^ (*11*). However, as noted above, we are estimating an alternative metric of the pattern effect than these studies based on comparison of different forcing scenarios; these numbers should thus be compared cautiously. We note that our results are consistent with the sliding window method applied to the same time series (fig. S3) (*8*, *11*), but that this latter method has drawbacks (Methods).

**Fig. 3.**
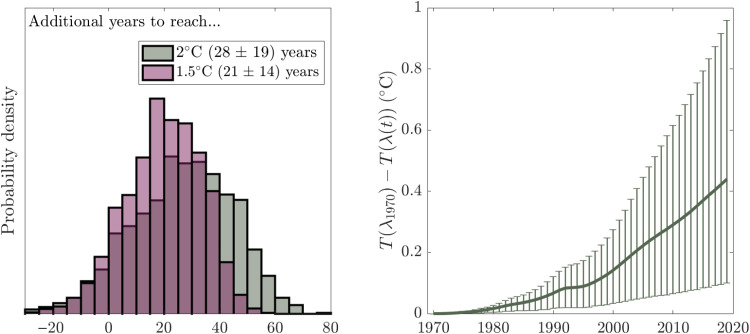
Differences in years to reach warming levels and in recent warming due to the diagnosed change in the climate feedback. (**Left**) Histogram of additional years necessary to reach 1.5° or 2°C under a 1%-per-year increase in atmospheric CO_2_ concentrations using the 2019 values of λ versus the 1970 values of λ in a simple energy balance model (Eq. 1). Note that as described in the text, this analysis is only illustrative to indicate the impact of the observed trend in λ from 1970 to 2019, and only the 1970 and 2019 values are compared here without making assumptions about the future extrapolation of the observed trend. (**Right**) Difference in *T* as a function of time between a scenario with observed λ trend versus constant 1970 λ value.

One way to estimate the impact of this trend is in terms of the time taken to reach a certain warming threshold, such as those laid out in the Paris agreement (*29*). To this end, we compare the time taken to reach 1.5° and 2°C for the 1970 and 2019 values of λ (Methods; Fig. 3 calculations). Under the idealized scenario where atmospheric CO_2_ concentrations increase 1% each year (*30*), this scenario results in a substantial difference in the time taken to cross these temperature thresholds; in a world with the 2019 λ value, it takes 21 ± 14 (28 ± 19) additional years to reach 1.5°C (2°C) than in a world with the 1970 value (Fig. 3, left). While this case is an idealized scenario and calculation, this difference demonstrates the importance of understanding and predicting the evolution of λ in recent and coming years. Similarly, we estimate that if λ had remained at its 1970 value for 1970–2019, an additional ∼0.4°C (66% range, 0.1 to 1.0) warming would have occurred by 2019 (Fig. 3, right) in addition to the ∼1.2°C that has occurred since 1900.

**Fig. 4.**
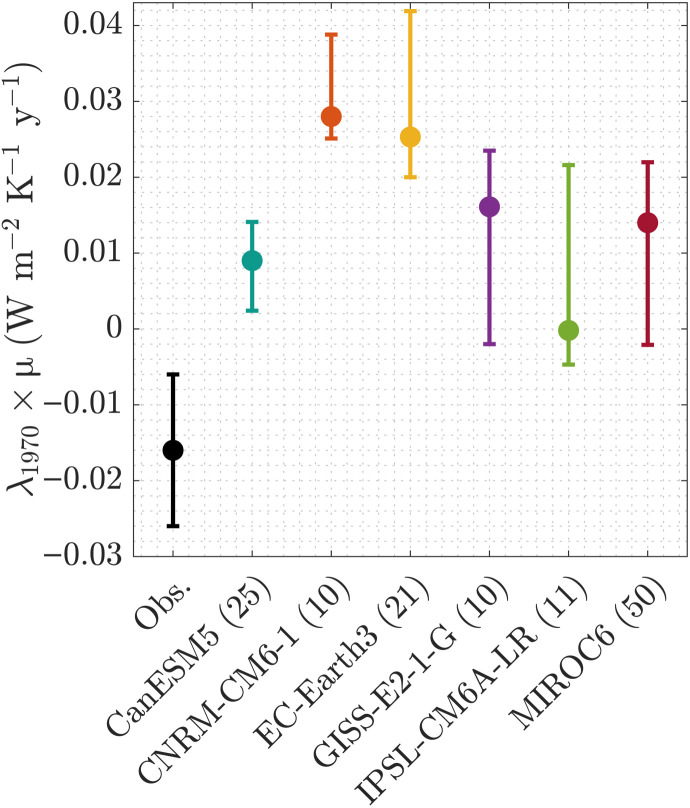
Median and 66% range of the trend of the climate feedback [i.e., μ × λ_1970_ (W m^−2^ K^−1^)] for the historical records (black) and their analogs from historical simulations of six climate models (color). Ensemble size for the climate models is given in parentheses.

We repeated our analysis of the historical time series with time series of model output of ensembles of historical simulations from six ESMs from Coupled Model Intercomparison Project phase 6 (CMIP6) (*30*) spanning a range of climate sensitivities. The ESM λ trends are either of the opposite sign to the observed trend or consistent with zero (Fig. 4) because many of the climate models do not capture observed surface warming patterns (*14*, *15*).

On the basis of observations alone, with minimal reference to climate models, our analysis exposes the substantial negative trend in the climate feedback over recent decades. Other work attributes this trend to changing patterns of sea surface warming (*5*, *7*). It remains a substantial challenge to understand this pattern effect and the evolution of climate feedback, and addressing that challenge is of paramount importance for climate projections.

## METHODS

### Theory

We begin with the energy balance equation, which states that the rate of warming of Earth’s surface is proportional to its net energy imbalance at the top of the atmosphere, here approximated by the net rate of surface and ocean heat uptake

$$\eta \dot{T}(t)=F(t)+\lambda (t)T(t)-H(t)$$ (1)

where *T* is the global mean surface temperature anomaly (K), η (W y m^−2^ K^−1^) is the heat capacity of the layer represented by *T*, *F* is the radiative forcing (W m^−2^), λ is the climate feedback (W m^−2^ K^−1^), and *H* is the heat uptake in the ocean below the layer represented by *T* (W m^−2^). Note that different authors use different sign conventions for λ; here, a stable climate has a negative λ. Here, we are interested in the evolution of the climate feedback λ(*t*). We approximate this evolution with the ansatz λ(*t*) = λ_1970_(1 + μ*t*); for simplicity, *t* is set to zero at 1970. We choose 1970 because both ocean heat content and global mean surface temperature increase very little before 1970 compared with uncertainty and interannual variability. Ocean heat content in particular before 1970 is very uncertain and sparsely observed. Inserting this ansatz and integrating both sides of this equation yields

$$\eta T(\tau )-\eta T(1970)=\int_{1970}^{\tau }F(t)\;dt+\lambda_{1970}\int_{1970}^{\tau }(1+\mu t)T(t)\;dt-\int_{1970}^{\tau }H(t)\;dt$$

We then define the integrals

$$F(\tau )=\int_{1970}^{\tau }F(t)\;dt,R(\tau )=-\int_{1970}^{\tau }\lambda (t)T(t)\;dt,H(\tau )=\eta [T(\tau )-T(1970)]+\int_{1970}^{\tau }H(t)\;dt$$

such that F is the cumulative energy fluxed to Earth’s surface via radiative forcing, R is the cumulative energy it has fluxed back to space, and H(τ) is the cumulative energy stored in the ocean and Earth’s surface. These last two are combined for two reasons: (i) The energy stored as warming of the Earth’s surface boundary layer, η*T*, is predominantly stored in the upper ocean. (ii) Observational records of ocean heat content cannot distinguish between the portion of energy storage in the ocean, which corresponds to this layer (η*T*) versus below this layer [$\int_{1970}^{\tau }H(t)\;dt$], so combining these terms is essential for comparison to observations. We can then use our ansatz and the defintion of R(τ) to define

$$T(\mu ,\tau )=\int_{1970}^{\tau }\;(1+\mu t)T(t)\;dt$$

which after substituting in these integral terms above and rearranging yields

$$F(\tau )-H(\tau )=-\lambda_{1970}T(\mu ,\tau )$$ (2)

which simply states that the amount of excess energy radiated back to space is equal to the excess energy added to the climate system by radiative forcing minus the amount stored in Earth’s system. The term on the right-hand side encodes the assumption that the climate feedback is changing with time at a constant rate. If the ansatz is valid and the correct μ is selected, this μ will capture the time dependence of λ and the slope of the regression of the left hand side against the right-hand side of the above equation will be constant in time, i.e., there will be no systematic behavior or curvature in the residuals of F(τ) − H(τ) regressed against T(μ, τ) (see Fig. 1).

The bulk of surface warming, and hence the bulk of the concentration of warming in very negative feedback regions, occurred since 1970 (*25*). Thus, the diagnosed difference between λ in 1970 versus 2019 is to some extent qualitatively comparable to the pattern effect, defined as the difference between the climate feedback in the absence versus the presence of historical warming in very negative feedback regions.

### Data

For *F*, we use the time series ensemble (2237 members) from the Intergovernmental Panel on Climate Change’s Working Group I contribution to the Sixth Assessment Report (*23*), which is available through 2019.

For *T*, we use the HadCRUT5 temperature record for global mean surface temperature because uncertainties being expressed as ensemble members make the propagation of uncertainty straightforward when integrating in time, and the HadCRUT5 ensemble captures the uncertainty across other temperature time series (*31*). *T* is defined as the temperature anomaly versus 1850–1900. HadCRUT5 is provided as a 200-member ensemble, with different dataset realizations that sample the measurement uncertainty, described in detail in (*22*); *T* in HadCRUT5 is a combination of surface air temperature over land and sea surface temperature elsewhere. From this ensemble a 2237-member ensemble is generated by estimating the multivariate mean and Gaussian covariance matrix from the 200-member ensemble and then randomly generating 2237-member ensemble with the same covariance properties and mean by sampling from a multivariate Gaussian probability distribution with this multivariate mean and covariance matrix. Repeating the analysis resampling directly from the 200-member ensemble had a negligible impact on the results. To test for the possible issue of Earth’s climate not being well represented as being in equilibrium in 1850–1900, we added 0.08 ± 0.03 W m^−2^ to *F* following (*32*) to correspond to the energy imbalance during the latter part of the 19th century; including this correction term had a negligible impact on the results. Note also that there was no relationship (Pearson, Spearman, and Kendall correlations <0.1) between μ or λ_1970_ and the initial temperature *T*(1970).

For H, we use the same method as in (*31*). The Japanese Meteorological Agency, (*26*), Cheng (*27*), and National Centers for Environmental Information (*28*) ocean heat content records are provided as ocean heat content over 0 to 2000 m. A 2237-member ensemble is generated from these by estimating the multivariate mean and Gaussian covariance matrix from the three time series and then randomly generating ensemble members with the same covariance properties and mean by sampling from a multivariate Gaussian probability distribution with this multivariate mean and covariance matrix. Years 1970 onward are considered because ocean heat content changes are more sparsely observed and uncertain before this year; furthermore, changes in both ocean heat content and temperature are very small over the years where ocean heat content data are available in a subset of these products before 1970 compared to both this uncertainty and interannual variability, indicating that there is little to no signal to extract.

### Primary analysis

To generate an estimate of λ_1970_ and μ for each *F*, *T*, and H ensemble pair, the following procedure is followed: we (i) sample a large range of λ_1970_ and μ values (we sampled these at sufficiently large ranges that no parameter estimates were at the boundaries of our sampled parameter space and, at a sufficiently fine resolution in parameter space, that increasing resolution by an order of magnitude did not change our results to the significant digits we report), (ii) calculate the residuals in Eq. 2 for these parameter values, and (iii) select the parameter values for which the linear regression has the lowest residual sum of squares. The linear ansatz is justified post hoc by performing a quadratic regression of the residuals against T(μ, τ); for 99% of ensemble members, the quadratic term of this regression is not significantly different from zero, and it is positive for 57% of ensemble members and negative for the other 43%. This indicates that the assumption that λ changes constantly in time successfully captures the temporal variation in λ.

### Sliding window method

Changes in λ over time have been studied in climate model simulations (particularly atmospheric simulations with prescribed sea surface temperatures) by regressing the change of global annual mean radiative response *dR* against surface air temperature change *dT* over a sliding 30-year window, e.g., (*11*). This method thus estimates a differential climate feedback (*20*). We performed the same analysis on the historical time series, estimating *dR* as *d*(*F* − *H*), with the standard 30-year window size. Figure S3 shows that this method agrees with our main result in Fig. 2. However, it gives larger uncertainties; is dependent on the ad hoc choice of window size, and can only provide estimates for the central 20 years of the time series, over which period no significant trend in λ can be detected from either method, and the use of a shorter sliding window produces estimates with large uncertainties and implausible fluctuations.

### Null hypothesis

The time evolution of λ is tested initially by performing the primary analysis described above with μ = 0. To test for systematic behavior in the residuals of the μ = 0 model, a quadratic regression of Eq. 2 with μ = 0 is performed for each ensemble member. For 92% of ensemble members the quadratic term is positive—i.e., F(τ) − H(τ) increases superlinearly with T(0, τ)—indicating that μ is significantly positive and a necessary parameter. We demonstrate this further by comparing the models with μ ≠ 0 and μ = 0 in terms of their AIC (*33*), the difference of which between two models estimates the difference in model quality. Figure S2 shows that for 91% of ensemble members, the ΔAIC values are negative, meaning the μ ≠ 0 model is a better description of the data even after being penalized for having an additional parameter. Similarly, we see no systematic behavior in the residuals of the main regression, indicating that unlike the μ = 0 case, there is no systematic behavior in the data that our λ = λ_1970_(1 + μ*t*) ansatz does not capture, although of course there are multiannual fluctuations that such a simple model cannot be expected to explain. Last, fig. S2 also shows that 92% of estimates of the trend in λ are negative, indicating that μ is significantly different from zero.

### Earth system models

We perform our primary analysis on ensembles of historical simulations using six ESMs for which global *F*, *T*, and top-of-atmosphere energy imbalance are available, whose time integral is approximately equal to H, for which we therefore use the cumulative integral noted N. The ESMs we use are the following: CanESM5 (*n* = 25 realizations), CNRM-CM6-1 (*n* = 10), EC-Earth3 (*n* = 21), GISS-E2-1-G (*n* = 10), IPSL-CM6A-LR (*n* = 11), and MIROC6 (*n* = 50), obtained via the CMIP6 archive (*30*). We append the *F*, *T*, and N estimates from these model realizations’ Shared Socioeconomic Pathway 2-4.5 simulations for 2015–2019, because historical *F* is only available up until 2014, but excluding years 2015–2019 had a negligible impact on the results. We also obtained five realizations from HadGEM3-CG31-LL, one from GFDL-CM4, and three from NorESM2-LM, but these are not included in Fig. 4 because only one of these nine realizations (a HadGEM3-CG31-LL realization where λ_1970_ × μ = + 0.02 W m^−2^ y^−1^ K^−1^) was within the range of the 2237 estimates from the observational historical ensemble, while the rest lie outside the *y*-axis range of Fig. 4.

### Figure 3 calculations

To estimate the difference in years taken to surpass 1.5° or 2°C in a world that has the 1970 parameter values versus one that has the 2019 values (each as time-invariant constant values), a 1% scenario is performed using each ensemble member’s (i) λ_1970_ value versus (ii) its climate feedback in 2019, i.e., λ_1970_(1 + 49μ). Under the 1% scenario, atmospheric CO_2_ concentrations increase by 1% per year, which, under the assumption of logarithmic forcing (*34*), results in a linear increase in *F* from zero until it reaches *F*_2×CO_2__ ∼ *N*(4.0,0.3) W y m^−2^ after 70 years (*9*). A random value of *F*_2×CO_2__ is sampled from *N*(4.0,0.3) for each ensemble member. We use the time-mean ocean heat uptake efficiency values, κ = 0.58 ± 0.08 W m^−2^ K^−1^, estimated in a similar fashion to our primary analysis for 1970–2019 (*31*) to simulate ocean heat uptake as *H*(*t*) = κ*T*(*t*); κ values for each ensemble member are drawn form a *N*(0.58,0.08) distribution. We use η values corresponding to the assumption that the surface layer represented by *T* has a heat capacity equal to the ocean’s mixed layer, sampling from a *N*(9.67,0.8) W y m^−2^ K^−1^ distribution following the calculation in (*35*). Note that this η estimate is a conservative upper limit and that reducing the η estimate to ∼0 had a negligible impact on the results. Using these values for *F*(*t*), η, λ, and κ, we simulate *T* using Eq. 1, find the year at which *T* > 1.5^∘^C and *T* > 2^∘^C for the 1970 and 2019 parameter values, and plot the difference between these in Fig. 3. Note that this is a heuristic metric and is only intended to illustrate the potential impact of the change in λ diagnosed here. To estimate the difference in *T* resulting from the trend in λ over the period 1970–2019, Eq. 1 is simulated using the historical ensembles’ *T*(1970) values and *F* time series, the same η and κ values as above, and either a fixed λ = λ_1970_ or the time-evolving λ = λ_1970_(1 + μ*t*). The right panel of Fig. 3 shows the difference between these two λ cases’ *T* evolutions. This difference therefore approximates the additional warming from 1970 to 2019 averted due to the increase in λ over this period. Note that when the historical ensembles’ *H* time series are used instead of a constant κ value, this difference is larger, with a median of 0.6°C (66% range, 0.1 to 1.4).
