# Supplementary material for: Energy budget diagnosis of changing climate feedback
Source: Sci Adv. 2023 Apr 21;9(16):eadf9302. doi: 10.1126/sciadv.adf9302 (PMC10121158; doi:10.1126/sciadv.adf9302)
Supplement: Supplementary file 1 — Figs. S1 to S3 [file sciadv.adf9302_sm.pdf]

Science Advances
AAAS

Supplementary Materials for

Energy budget diagnosis of changing climate feedback

B. B. Cael *et al.*

Corresponding author: B. B. Cael, cael@noc.ac.uk

Sci. Adv. **9**, eadf9302 (2023)
DOI: 10.1126/sciadv.adf9302

This PDF file includes:

Figs. S1 to S3

Supplementary figures

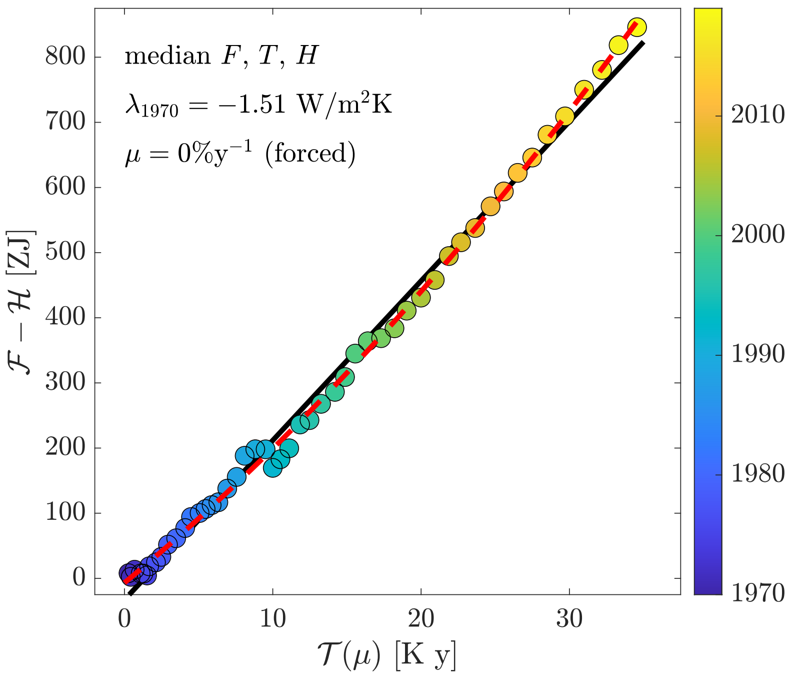

Supplementary Figure 1: As Figure 1 but with μ forced to equal zero. The red line indicates the best-fit quadratic polynomial to the scatterpoints, for which the quadratic term is significantly different than zero and the quadratic polynomial is a significantly better fit than the linear fit ($p \ll 0.01$), neither of which is the case for the $\mu \neq 0$ case shown in Figure 1.

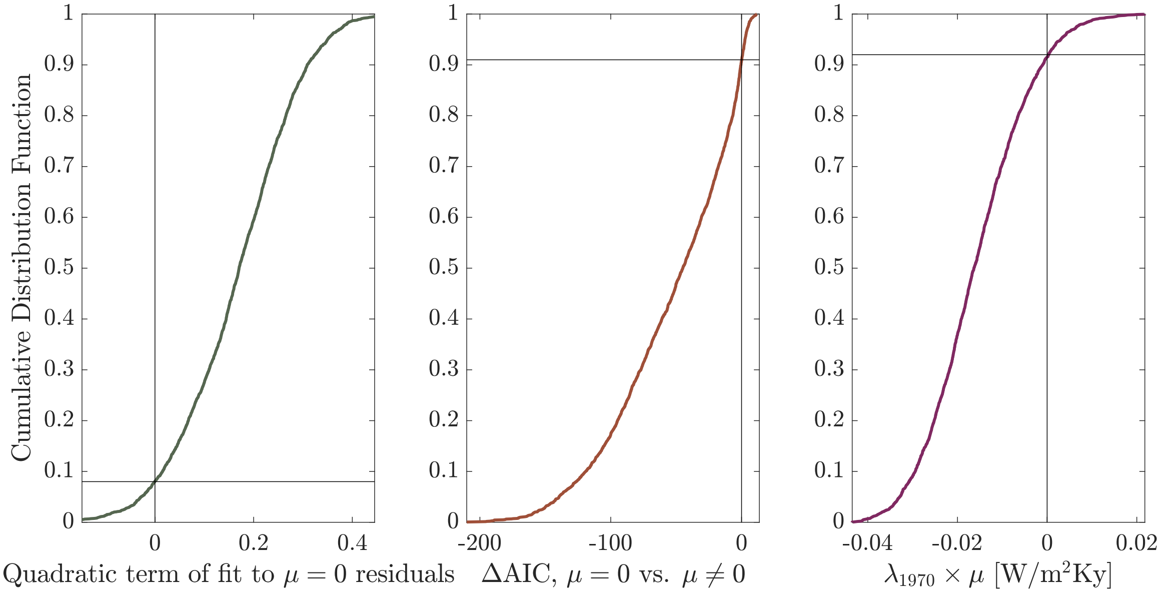

Supplementary Figure 2: Cumulative distribution functions across ensemble members of – Left: the value of the quadratic term in a quadratic polynomial fit to the residuals of the $\mu = 0$ model. Center: the difference in the Akaike Information Criterion for the ansatz used here versus a 'linear' model with a constant climate feedback. Negative ΔAIC values indicate that the ansatz used here is a better description of the historical time series. Right: the trend in λ from 1970–2019 diagnosed with the $\mu \neq 0$ model. In each case the black lines indicate the fraction of ensemble members for which the quantity on the x-axis is negative.

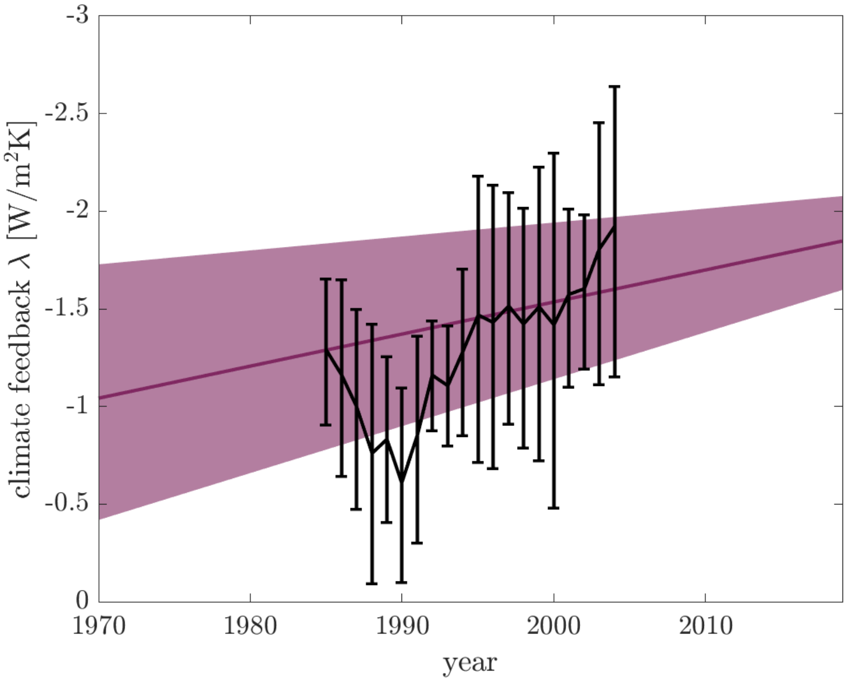

Supplementary Figure 3: As Figure 2 but with climate feedback as estimated by regression of dR against dT over a sliding window of 30 years, as in [11], superimposed. Error bars in each case represent 66% confidence interval. Note that for all years for which the sliding window method can estimate λ, our median estimate is within the sliding window method's 90% confidence interval.
